# Supplementary material for: Stepping forward: A study protocol for developing and validating a Malaysian diabetic foot self-care practice assessment instrument
Source: PLoS One. 2025 Dec 31;20(12):e0337754. doi: 10.1371/journal.pone.0337754 (PMC12755747; doi:10.1371/journal.pone.0337754)
Supplement: S1 File — (PDF) [file pone.0337754.s001.pdf]

Annex 1: Data extraction form for literature review (will be built in Excel sheets)

COLUMN HEADING	INPUT TYPE	OPTIONS, IF APPLICABLE
Document title	Open text	N/A
Publication year	Open text	N/A
Authors	Open text	N/A
Type of document	Dropdown	Instrument development/validation, Knowledge, Attitude, Practice questionnaires
Country	Open text	
Name of instrument	Open text	N/A
Domains	Open text	N/A
Number of items	Open text	N/A
Scoring	Open text	N/A
Scoring interpretation	Open text	N/A
Reliability and validity psychometrics	Open text	N/A
Additional comments	Open text	N/A

Annex 2: Structured Evaluation Criteria Form for Content Validation

VALIDATION OF THE MALAYSIAN DIABETIC FOOT SELF-CARE ASSESSMENT INSTRUMENT
Dear Experts,<br><br>This evaluation form contains 5 domains to evaluate the draft of the instrument developed to assess the diabetic foot self-care of diabetic patients referred for foot care services. We need your expert judgement on the domains stated below. Your review should be based on the definition and relevant terminologies that are provided to you. Please be as objective and constructive as possible in your review and use the rating scale given. Please check (✓) only one from the selection.

**DOMAIN 1: SUITABILITY/RELEVANCE OF THE ITEMS**<br><br>**Definition:** The questions are designed to determine the self-care practices that are relevant and important to be measured in the Malaysian setting<br><br>**Degree of relevance:**<br>1 = the item is <u>not relevant</u> to measure diabetic foot self-care<br>2 = the item is <u>somewhat relevant</u> to measure diabetic foot self-care<br>3 = the item is <u>quite relevant</u> to measure diabetic foot self-care<br>4 = the item is <u>highly relevant</u> to measure diabetic foot self-care	
**Items**	**Relevance**
Item 1 :	1 [ ] 2 [ ] 3 [ ] 4 [ ]
Feedback:	
Item 2 :	1 [ ] 2 [ ] 3 [ ] 4 [ ]
Feedback:	
.	
.	
**DOMAIN 2: ORGANIZATION AND PRESENTATION OF ITEMS**	
**Definition**	**Scale**
The items are organised and presented in logical and sequential manner	Poor [ ] Fair [ ] Good [ ] Very good [ ]
Feedback:	

**DOMAIN 3: CLARITY OF THE ITEMS**<br><br>**Definition:** The vocabulary level of the items suits the level of respondents. The items are written in a clear and understandable manner.	
**Definition**	**Scale**
Item 1:	Poor Fair Good Very good<br>[ ] [ ] [ ] [ ]
Feedback:	
Item 2:	Poor Fair Good Very good<br>[ ] [ ] [ ] [ ]
Feedback:	
.	
.	
**DOMAIN 4: ADEQUATENESS & SUITABILITY OF THE SCORING USED**	
**Definition**	**Scale**
The number of items is representative enough to assess the diabetic foot self-care	Poor Fair Good Very good<br>[ ] [ ] [ ] [ ]
Feedback:	
The scoring mechanism is easily understood to be administered for daily use	Poor Fair Good Very good<br>[ ] [ ] [ ] [ ]
Feedback:	
Items XX are suitable to be placed under Section A	Poor Fair Good Very good<br>[ ] [ ] [ ] [ ]
Feedback:	
The scoring options for Section A is appropriate	Poor Fair Good Very good<br>[ ] [ ] [ ] [ ]
Feedback:	
.	
.	
**DOMAIN 5: ATTAINMENT OF PURPOSE**	
**Definition**	**Scale**
The instrument as a whole fulfils the objective for which it was constructed	Poor Fair Good Very good<br>[ ] [ ] [ ] [ ]
**OTHER FEEDBACK:**	
……………………………………………………………………………………………<br><br>……………………………………………………………………………………………<br><br>……………………………………………………………………………………………	

Annex 3: Structured Form for Face Validation

CRITERIA TO RATE	ANSWER
Instructions given on the instrument is clear	[ ] Yes [ ] No
Language used is easily understood	[ ] Yes [ ] No
The questions asked are clear	[ ] Yes [ ] No
The structure of the sentences is correct	[ ] Yes [ ] No
Difficulty level of the items asked is reasonable	[ ] Yes [ ] No
The number of items in relation to the supposed purpose of the instrument is reasonable	[ ] Yes [ ] No
The items in the instrument have adequate flow to guide understanding	[ ] Yes [ ] No
The scoring mechanism is easy and clear to use	[ ] Yes [ ] No
**ADDITIONAL FEEDBACK**	
For questions answered “no”, please do elaborate and suggest improvements<br>……………………………………………………………………………………………<br>……………………………………………………………………………………………<br>……………………………………………………………………………………………	

Annex 4: Calculation Table for Content Validity Index / Face Validity Index

**Question**	**Expert 1**	**Expert 2**	**Expert 3**	**Expert 4**	**Expert 5**		**Experts in agreement**	**I-CVI**	**Universal agreement (UA)**
**Q1**									
**Q2**									
**Q3**									
**Q4**									
**Q5**									
**Q6**									
**Q7**									
**Q8**									
**Q9**									
**Q10**									
**Q11**									
**Q12**									
**Q13**									
**Q14**									
**Q15**									
**Q16**									
**.**									
**.**									
							**S-CVI/ Ave**		
**Proportion relevance**							**S-CVI/ UA**		
	**Average proportion of questions rated as relevant/good across the 5 experts**					**S-CVI/ Ave**			

***** Headings of the table will be amended accordingly for Face Validity Index
