# Supplementary material for: Stepping forward: A study protocol for developing and validating a Malaysian diabetic foot self-care practice assessment instrument
Source: PLoS One. 2025 Dec 31;20(12):e0337754. doi: 10.1371/journal.pone.0337754 (PMC12755747; doi:10.1371/journal.pone.0337754)
Supplement: Attachment — Submitted filename: Response to Reviewers.docx [file pone.0337754.s002.docx]

**RESPONSE TO REVIEWERS**

PONE-D-25-16530 - Stepping Forward: A Study Protocol for Validating a Malaysian Diabetic Foot Self-care Practice Assessment Instrument

| **NO.** | **COMMENTS** | **RESPONSE** |
| --- | --- | --- |
| **Journal Requirements / Editor’s Comments** | | |
| 1 | Please ensure that your manuscript meets PLOS ONE's style requirements, including those for file naming.  The PLOS ONE style templates can be found at  [https://journals.plos.org/plosone/s/file?id=wjVg/ PLOSOne_formatting_sample_main_body.pdf](https://journals.plos.org/plosone/s/file?id=wjVg/%20PLOSOne_formatting_sample_main_body.pdf) and [https://journals.plos.org/plosone/s/file?id=ba62/ PLOSOne_formatting_sample_title_authors_affiliations.pdf](https://journals.plos.org/plosone/s/file?id=ba62/%20PLOSOne_formatting_sample_title_authors_affiliations.pdf) | Thank you for the guidance. Where applicable and required, the manuscript has been reformatted to align with the PLOS ONE style requirements. |
| 2 | We note that the grant information you provided in the ‘Funding Information’ and ‘Financial Disclosure’ sections do not match.  When you resubmit, please ensure that you provide the correct grant numbers for the awards you received for your study in the ‘Funding Information’ section. | Thank you for highlighting the discrepancy.   - I have amended the ‘Funding Information’ as instructed to ensure it is accurate to reflect the following:   This research is funded by the Ministry of Health Malaysia Research Grant [Grant reference number: KKM.400-4/2/30 Jld.2 (52)]. The funding body is not involved in the study design, data collection and analysis, publication decision, or manuscript preparation.   - Nevertheless, I am **unable to amend the 'Financial Disclosure' section**, as I **no longer have access** to it, possibly because the journal has **not granted permission** for further **edits** to that section |
| 3 | Your ethics statement should only appear in the Methods section of your manuscript. If your ethics statement is written in any section besides the Methods, please move it to the Methods section and delete it from any other section.  Please ensure that your ethics statement is included in your manuscript, as the ethics statement entered into the online submission form will not be published alongside your manuscript. | Thank you for highlighting this important aspect.   - We have now ensured that the full ethics statement is included exclusively in the “Methods” section (lines 377–389: marked up version), as advised. |
| 4 | We note that you have indicated that there are restrictions to data sharing for this study. For studies involving human research participant data or other sensitive data, we encourage authors to share de-identified or anonymized data.  However, when data cannot be publicly shared for ethical reasons, we allow authors to make their data sets available upon request. For information on unacceptable data access restrictions, please see <http://journals.plos.org/plosone/s/data-availability#loc-unacceptable-data-access-restrictions>.  Before we proceed with your manuscript, please address the following prompts:   a) If there are ethical or legal restrictions on sharing a de-identified data set, please explain them in detail (e.g., data contain potentially identifying or sensitive patient information, data are owned by a third-party organization, etc.) and who has imposed them (e.g., a Research Ethics Committee or Institutional Review Board, etc.). b) Please also provide contact information for a data access committee, ethics committee, or other institutional body to which data requests may be sent. c) If there are no restrictions, please upload the minimal anonymized data set necessary to replicate your study findings to a stable, public repository and provide us with the relevant URLs, DOIs, or accession numbers. d) Please see <http://www.bmj.com/content/340/bmj.c181.long> for guidelines on how to de-identify and prepare clinical data for publication. For a list of recommended repositories, please see <https://journals.plos.org/plosone/s/recommended-repositories>. e) You also have the option of uploading the data as Supporting Information files, but we would recommend depositing data directly to a data repository if possible.   Please update your Data Availability statement in the submission form accordingly. | Thank you for your detailed guidance on data sharing requirements.   - As this is a study protocol, no data have been generated or analysed at this stage. However, we acknowledge the importance of data availability and have updated the “Data Availability Statement” section in the manuscript and submission form to reflect the following: - As this is a study protocol, no datasets have been generated or analysed at this stage. Upon completion and publication of the study, de-identified relevant research data will be made available upon reasonable request from the corresponding author or the Head of Centre for Biostatics & Data Repository, National Institutes of Health, Ministry of Health Malaysia, and subject to approval by the Director General of Health, Malaysia. (as elaborated from lines 370-376: marked up version) |
| 5 | In the online submission form, you indicated that data used in this study is not publicly available due to confidentiality restrictions. However, data can be accessed upon reasonable request and subject to approval from the corresponding author or the Head of Centre for Biostatics & Data Repository, National Institutes of Health, Ministry of Health Malaysia on reasonable grounds for the request and with the permission from the Director General of Health, Malaysia.  All PLOS journals now require all data underlying the findings described in their manuscript to be freely available to other researchers, either 1. In a public repository, 2. Within the manuscript itself, or 3. Uploaded as supplementary information.  This policy applies to all data except where public deposition would breach compliance with the protocol approved by your research ethics board. If your data cannot be made publicly available for ethical or legal reasons (e.g., public availability would compromise patient privacy), please explain your reasons on resubmission and your exemption request will be escalated for approval. |  |
| **Reviewer’s Comments** | | |
| 6 | Is the protocol technically sound and planned in a manner that will lead to a meaningful outcome and allow testing the stated hypotheses?  The manuscript should describe the methods in sufficient detail to prevent undisclosed flexibility in the experimental procedure or analysis pipeline, including sufficient outcome-neutral conditions (e.g. necessary controls, absence of floor or ceiling effects) to test the proposed hypotheses and a statistical power analysis where applicable. As there may be aspects of the methodology and analysis which can only be refined once the work is undertaken, authors should outline potential assumptions and explicitly describe what aspects of the proposed analyses, if any, are exploratory.  Reviewer #1: Partly | Thank you for your valuable feedback. We understand and appreciate your concerns regarding the use and adaptation of existing global instruments.   - In response, we have carefully reconsidered our approach and have revised the protocol accordingly. - The proposed study will no longer involve the adoption or adaptation of any existing scales; instead, we intend to develop a new Malaysian-specific instrument. - To inform/guide this development, the comprehensive literature review will examine the strengths and limitations of previously established instruments. - Insights gained from this review will guide the design of our instrument, aligning it with the specific needs of Malaysian stakeholders while also ensuring internationally recognised constructs to support cross-cultural comparability. - We are also mindful of potential proprietary and licensing issues that may arise from using established tools. For example, the Morisky Medication Adherence Scale, which was previously adopted and adapted in our country, later required licensing fees that became too costly for sustained national use and was eventually discontinued. - Developing a contextually relevant instrument from the outset will help us avoid such challenges and ensure broader accessibility for future nationwide implementation. - We remain committed to ethical research practices and will ensure that content drawn from existing instruments are appropriately acknowledged and cited. - Additionally, we have expanded the methodological section to provide greater detail on our instrument development process. This is intended to enhance transparency and support replicability of the study by researchers in other countries with similar contextual needs. - Amended in manuscript (marked up version) in the following lines: - 28-31 - 92-95 - 97-99 - 120-125 - 147-151 - 158-165 - 174-178 - 256-257 - 312-315 - 321-322 - 326-328 - 391-392 - 421-423 |
| 7 | Is the methodology feasible and described in sufficient detail to allow the work to be replicable?   Descriptions of methods and materials in the protocol should be reported in sufficient detail for another researcher to reproduce all experiments and analyses. The protocol should describe the appropriate controls, sample size calculations, and replication needed to ensure that the data are robust and reproducible.  Reviewer #1: No |  |
| 8 | Reviewer #1: I see ethical problems with the use of items from other global scales and, worse, with the adaptation and modification of items from these scales without prior authorization from the authors of the original scales. I suggest taking the best scale and, with the authorization of the authors of the original scale, translating it into the language of your country. I recommend TO REFUSE to publish this article in its current form. |  |
| 9 | Have the authors described where all data underlying the findings will be made available when the study is complete?  The [PLOS Data policy](https://track.editorialmanager.com/CL0/https:%2F%2Fjournals.plos.org%2Fplosone%2Fs%2Fmaterials-and-software-sharing/1/010f0196a3b5b749-a35e9b7d-1a42-4c0f-85d0-cd738734ad74-000000/4lUmGE-RhIV_05iKO3Fk9eFJlKjVA_bn4YCfYbT8Ngg=209) requires authors to make all data underlying the findings described in their manuscript fully available without restriction, with rare exception, at the time of publication. The data should be provided as part of the manuscript or its supporting information, or deposited to a public repository. For example, in addition to summary statistics, the data points behind means, medians and variance measures should be available. If there are restrictions on publicly sharing data—e.g. participant privacy or use of data from a third party—those must be specified.  Reviewer #1: No | Thank you for highlighting this important point.   - As this is a study protocol, no datasets have been generated or analysed at this stage. - However, we have revised the manuscript under the “Data Availability Statement” clarifying that, upon completion and publication of the study, de-identified relevant data will be made available upon reasonable request from the corresponding author or the Head of the Centre for Biostatistics & Data Repository, National Institutes of Health, Ministry of Health Malaysia, and subject to approval by the permission from the Director General of Health, Malaysia. (as elaborated from lines 370-376: marked up version) |
| 10 | While revising your submission, please upload your figure files to the Preflight Analysis and Conversion Engine (PACE) digital diagnostic tool, [https://pacev2.apexcovantage.com/](https://track.editorialmanager.com/CL0/https:%2F%2Fpacev2.apexcovantage.com%2F/1/010f0196a3b5b749-a35e9b7d-1a42-4c0f-85d0-cd738734ad74-000000/scYIb7MehgyelSyM7eJObJWHJrQ_dD8ypsH-o_d9aKU=209). PACE helps ensure that figures meet PLOS requirements.  To use PACE, you must first register as a user. Registration is free. Then, login and navigate to the UPLOAD tab, where you will find detailed instructions on how to use the tool. If you encounter any issues or have any questions when using PACE, please email PLOS at [figures@plos.org](mailto:figures@plos.org). Please note that Supporting Information files do not need this step. | Thank you for the instructions.   - All figure files have been uploaded to the Preflight Analysis and Conversion Engine (PACE) and have passed the required checks. - We have ensured that the figures meet PLOS formatting requirements. - Fig 1, Fig 2 and Fig 3 have been uploaded as .tif files, labelled accordingly (label of figure). |
