# Supplementary material for: Stepping forward: A study protocol for developing and validating a Malaysian diabetic foot self-care practice assessment instrument
Source: PLoS One. 2025 Dec 31;20(12):e0337754. doi: 10.1371/journal.pone.0337754 (PMC12755747; doi:10.1371/journal.pone.0337754)
Supplement: Attachment — Submitted filename: Response to Reviewers (Cycle 2).docx [file pone.0337754.s003.docx]

**RESPONSE TO REVIEWERS CYCLE 2**

PONE-D-25-16530 - Stepping forward: A study protocol for developing and validating a Malaysian diabetic foot self-care practice assessment instrument

| **NO.** | **COMMENTS** | **RESPONSE** |
| --- | --- | --- |
| **Journal Requirements / Editor’s Comments** | | |
| 1 | - Please review your reference list to ensure that it is complete and correct. - If you have cited papers that have been retracted, please include the rationale for doing so in the manuscript text, or remove these references and replace them with relevant current references. - Any changes to the reference list should be mentioned in the rebuttal letter that accompanies your revised manuscript. - If you need to cite a retracted article, indicate the article’s retracted status in the References list and also include a citation and full reference for the retraction notice. | - We thank the reviewer for this important reminder. - In response, we undertook a thorough review of the entire reference list to ensure that every source cited is complete, verifiable, and fully compliant with PLOS ONE’s rigorous publication standards. - We confirm there were no retracted articles in the reference list. - Nevertheless, 3 references were removed as they may not meet the required standards for robust peer-review, retrievability, or scientific credibility:  a. Goweda et al. (2017) was removed because it is not widely indexed and its content is sufficiently covered by stronger, peer-reviewed sources already included in the manuscript. b. AHS et al. (2018) was removed due to lack of indexing and unclear peer-review status.  - To uphold the scientific foundation of this section, we actively replaced it with a more credible, peer-reviewed source (Sulistyo et al., 2018, *Journal of Research in Nursing*), which reports on the same instrument and context.  c. Quadri et al. (2013) was removed as it is a conference abstract only, with no peer-reviewed full-text version available, and therefore does not meet PLOS ONE’s citation requirements.  - By carefully verifying, removing, and replacing these references, we have taken deliberate steps to strengthen the scholarly rigor and integrity of the manuscript. - We have ensured that all references meet the journal’s expectations for transparency, retrievability, and quality. |
| **Reviewer’s Comments** | | |
| 2 | Is the protocol technically sound and planned in a manner that will lead to a meaningful outcome and allow testing the stated hypotheses?  The manuscript should describe the methods in sufficient detail to prevent undisclosed flexibility in the experimental procedure or analysis pipeline, including sufficient outcome-neutral conditions (e.g. necessary controls, absence of floor or ceiling effects) to test the proposed hypotheses and a statistical power analysis where applicable. As there may be aspects of the methodology and analysis which can only be refined once the work is undertaken, authors should outline potential assumptions and explicitly describe what aspects of the proposed analyses, if any, are exploratory.  Reviewer #2: Partly | - We have addressed these methodological aspects through our responses to the specific comments provided and have revised the manuscript accordingly to ensure sufficient detail, clarity, and transparency. |
| 3 | Review Comments to the Author:  The aim of this study protocol is describe the development and validation of a Malaysian diabetic foot self-care practice assessment instrument. In this revised version, the recommendations previously made have been taken into account and the manuscript has been improved, and I have only some minor comments. | - We thank the reviewer for acknowledging the improvements and appreciate the constructive feedback provided throughout the review process. - We will address the remaining minor comments accordingly to further strengthen the manuscript. |
| 4 | Review Comments to the Author:   - At the end of the introduction, the aim and/or objectives of the study should be clearly defined. - As the proposed study will first develop and then validate a national instrument, both aspects should also be mentioned in the objectives. | - We thank the reviewer for this valuable suggestion. - In response, we have revised the study objectives in both the Abstract and Introduction sections to explicitly state that the study involves both the development and validation of the national instrument. - Lines 23, 25, 26 (Abstract) - Line 130 (Introduction) - We believe this amendment clarifies the full scope of the study as intended. |
| 5 | Review Comments to the Author:   - As the authors have changed the structure of the study compared to the first version of the manuscript, they should also change the title. | - We thank the reviewer for this constructive suggestion. We concur that the revised structure of the study should be clearly reflected in the title. - Accordingly, we have amended the title to: “Stepping Forward: A Study Protocol for Developing and Validating a Malaysian Diabetic Foot Self-care Practice Assessment Instrument” and the short title to “Developing and validating a Malaysian diabetic foot self-care practice assessment instrument” - These revised titles more accurately represent the comprehensive scope of the study, which now encompasses both the development and validation phases of the proposed national instrument. |
| 6 | Review Comments to the Author:   - Study type and design - please clearly state that the first stage is the development of the instrument and the second is the validation study. | - We thank the reviewer for this helpful suggestion. - We have revised the manuscript to clearly state that Phase 1 involves the development of the instrument and Phase 2 involves its validation through a cross-sectional study design. - Lines 144-146 - Lines 383-386 |
| 7 | Review Comments to the Author:   - Patients will be involved in the second phase of the study - validation through a cross-sectional study. - However, the new instrument will be self-administered by the patients, so the patients should be involved already in the development process - from the definition of the topics/items of the instrument and the content validation, patients should be involved in the expert group, in addition it may be appropriate to conduct a qualitative survey among patients. | - We sincerely thank the reviewer for highlighting this important point. - We agree that incorporating patient perspectives is crucial to ensure that the instrument is clear, relevant, and practical for its intended users. - In Phase 1, the domains and items will be systematically drafted based on established national diabetic foot care guidelines and robust international evidence. - This initial version will undergo expert content validation by a panel of healthcare professionals with extensive experience in diabetic foot care to ensure clinical relevance and accuracy. - To ensure that the instrument also meets patient needs and expectations, patients will be involved in the face validity process in Phase 1 through pre-testing and cognitive debriefing. - This will include: - A short, structured feedback form for patients - Cognitive debriefing interviews to gather in-depth feedback on wording, cultural appropriateness, and ease of use - We will carefully analyse this feedback to refine the instrument before proceeding to piloting and field-testing in Phase 2 : Lines 216–232 - This multi-step approach ensures that the instrument content is evidence-based, clinically sound, and meaningfully co-shaped by patient input, hence maximizing its practical usability and cultural appropriateness. |
